# Emerging implications of exosomes as mediators of cellular communication in potentially malignant oral lesions and head and neck cancers

**DOI:** 10.12688/f1000research.127368.1

**Published:** 2023-01-13

**Authors:** Monica Charlotte Solomon, Chetana Chandrashekar, Spoorti Kulkarni, Nisha Shetty, Aditi Pandey

**Affiliations:** 1 Department of Oral and Maxillofacial Pathology and Oral Microbiology, Manipal College of Dental Sciences, Manipal, Manipal Academy of Higher Education, Manipal, Karnataka, 576104, India

**Keywords:** Exosomes, miRNA, intercellular communication, tumor microenvironment, invasion and metastasis

## Abstract

Exosomes are a unique type of extracellular vesicles that contain a plethora of biological cargo such as miRNA, mRNA, long non-coding RNA, DNA, proteins and lipids. Exosomes serve as very effective means of intercellular communication. Due the presence of a lipid bilayer membrane, exosomes are resistant to degradation and are highly stable. This makes them easily identifiable in blood and other bodily fluids such as saliva. The exosomes that are secreted from a parent cell directly release their contents into the cytoplasm of a recipient cell and influence their cellular activity and function. Exosomes can also transfer their content between cancer cells and normal cells and regulate the tumor microenvironment. Exosomes play a vital role in tumor growth, tumor invasion and metastasis. Exosomes provide a multitude of molecular and genetic information and have become valuable indicators of disease activity at the cellular level. This review explores the molecular characteristics of exosomes and the role that exosomes play in the tumorigenesis pathway of potentially malignant oral lesions and head and neck cancers The application of exosomes in the treatment of oral cancers is also envisioned.

Exosomes are very small and can easily pass through various biological barriers, making them very good delivery vectors for therapeutic drugs as well as to selectively induce DNA’s mRNA and miRNAs into targeted cancer cells.

## Introduction

Exosomes are a form of extracellular vesicles (EVs) that were first described by Colombo *et al.* in 2014. ^1^ Exosomes are formed intracellularly, and the size of exosomes ranges from 30nm-150nm. The exosomes contain a plethora of biological entities such as proteins, lipids, DNA, microRNA, messenger RNA and long noncoding RNA enclosed in a lipid bilayer. ^2^^–^^5^

Exosomes originally form as intraluminal vesicles within the endosome and are released to the environment by fusion with the plasma membrane. ^1^ Exosomes are formed by the invagination of the endosomal plasma membrane during the transformation of an early endosome into a late endosome. The late endosomes, also known as microvesicular bodies, fuse with the cell membrane and release their contents into the extracellular environment and are now called exosomes. ^6^ The formation of exosomes depends on two different pathways; the first an endosomal sorting complex required for transport (ESCRT)-dependent mechanism, and an ESCRT- independent mechanism. ^7^ Several factors influence the release of exosomes into the extracellular environment, such as oxidative stress and hypoxia. In addition, drugs such as sitafloxacin, pentatrezole and fenoterol activate the production of exosomes. ^8^

Exosomes are an unique mode of intercellular communication for transferring bioactive cargo to recipient cells. ^9^ As exosomes are released into the extracellular compartment, they are richly available in most of the body’s fluids including saliva. The lipid bilayer membrane that forms around the exosome capsule protects their cargo from degradation and RNAse damage, thus allowing exosomes to be highly stable in circulation. ^10^

Exosomes play a vital role in transferring molecular mediators and promote cell-to-cell communication both locally and to distant sites. ^11^ When exosomes release the protein, signaling molecules, mRNA and miRNA into the cytoplasm of the target cells, this modifies the cell biology of that target cell. ^12^

The mRNA and the miRNA that are present in exosomes can be translated into proteins in the target cells that they enter, thereby transferring genetic information from one cell to another cell. ^13^

This review elucidates the molecular characteristic of exosomes and their implication in the biology of potentially malignant oral disorders and head and neck cancers.

## Characteristic features of exosomes

Zlotogorski *et al.* ^14^ elucidated the molecular characteristics of exosomes through several molecular techniques such as transmission electron microscopy, nanoparticle tracking, atomic force microscopy, ELISA and Western blot. In his experimental analysis he compared the morphological features and molecular characteristic of exosomes in oral fluids of healthy individual and oral cancer patients.

## Transmission electron microscopy

The ultrastructural features of the pellets that were obtained from saliva of healthy individuals and oral cancer patients are similar. They appear as round-shaped vesicles surrounded by bilayered membranes.

## Nanoparticle tracking analysis

The number of exosomes in saliva as determined by nanoparticle tracking analysis was 17.9 ±12.45E8 particles/ml in healthy individuals, while that in oral cancer patients was 36.0± 7.5E8 particles/ml. The average size of the nanoparticles in healthy individuals was 49.05 ± 32.87 nm and that of exosomes in oral cancer patients was 95.36±36.76 nm, which is much larger than that of healthy Individuals.

## Atomic force microscopy

The 3D topographical images of the nanoparticle in the Oral fluids of both healthy individuals and oral cancer patients appeared as circular bulging structures, but the height of the particles in oral cancer patients was larger than the height of those in healthy individuals.

## Enzyme linked immunosorbent analysis (ELISA)

The concentration of CD63 in the exosomes in the oral fluids was higher in oral cancer patients (234±79 pg/ml) compared with that of normal individuals (176±42.3pg/ml). The concentration of CD81 in exosomes was lower in OSCC (61.1±37 pg/ml) compared to that of healthy individuals (201±pg/ml). The concentration of CD9 in the exosomes of oral cancer was less (104.1±18 pg/ml) than that of healthy individuals (152.9±24.4pg/ml).

## Western blot

In oral cancer patients the exosomal pellets obtained from the oral fluids showed the presence of the glycosylated form of CD63 as a prominent band at 53Kd. With regard to CD81, the exosomes obtained from the oral fluids of oral cancer patients and healthy individuals showed a band at the expected 26 kDa area of the protein. The exosomes obtained from the oral fluid of both oral cancer patients and healthy individuals showed a specific band at 28kDa area to represent CD9. However, in oral cancer patients the intensity of the band was lower than that in healthy individuals.

Mathivanan *et al* also found a significant increase in the expression of CD63 and a decrease in the expression of CD9 and CD81 in exosomes present in the saliva of oral cancer patients compared to that of the exosomes in the saliva of healthy individuals. ^6^ Exosomes are recognized by the molecular biomarkers that they express; namely, CD63, CD9, CD81, Alix, TSG101 and hsp70. ^15^

Proteins that are encapsulated and transported by exosomes are responsible for regulating the fusion, migration, and adhesion to the target cells. These proteins are transmembrane proteins such as CD9, CD63, CD81 and CD82 molecular chaperones Hsp70 and Hsp90, and multi-capsule synthesis proteins TSG101 and ALIX. ^16^^–^^20^ The miRNAs in the exosomes are of higher concentration and greater stability while they are in circulation as they are within encapsulated vesicles. ^11^^,^^21^^,^^22^ The proteases and RNAases in circulation cannot act on the exosomal proteins; hence they have a longer half-life than the free molecules. ^19^ Exosomes are enriched in cholesterol, diglycerides, glycerophospholipids, phospholipids and sphinolipids or glyceromides ^23^

## Exosomes in oral potentially malignant disorders

Li *et al.* isolated mesenchymal stem cells from the clinical tissue samples of normal oral mucosal tissues, dysplastic oral lesions and oral squamous cell carcinoma tissue. The exosomes were isolated from the Mesenchymal stem cells and a microarray analysis of the exosomes showed that miR 8485 was differentially expressed in the three groups. The miRNA-8485, when transfected into dysplastic oral mucosal cell lines (DOK) as well as tongue squamous cell carcinoma cell line SCC15, caused rapid growth, promoted migration and invasion of the cells. ^24^

Wang *et al.* found that when MSC-EV-miR-185 was pasted onto buccal lesions in dimethylbenzanthracene (DMBA) induced Oral Potentially Malignant disorders (OPMD) model it remarkably attenuated the severity of inflammation and significantly decreased the incidence and the number of dysplastic characteristics in the OPMD tissue. Also, these cells showed a low immunohistochemical expression of PCNA and CD 31. By activating caspase 3 and 9 of the apoptotic pathway, the miR-185 targeted the Akt pathway. ^25^

## Exosomes in oral submucous fibrosis

Oral submucous fibrosis is a multifactorial precancer disorder that is caused by chewing areca nuts. Liu *et al.* isolated adipose-derived mesenchymal stem cell exosomes from the fibroblasts of oral submucous fibrosis patients and normal individuals. These ADSC-Exos were found to be positive for CD63. The mRNA expression levels of COLIA 1 and COLIA III were down regulated in fibroblasts that were stimulated with both TGF- Beta and ADSC-Exos in the culture media. Similarly, the expression of Collagen I and Collagen III was downregulated in fibroblasts that were treated with both TGF Beta and ADSC-exosomes. The ADSC-exosomes inhibited the p38 MAPK signalling pathway and reduced the expression of collagen I and Collagen III.

However, the expression levels of matrix metalloproteinase (MMP)1 and MMP3 were significantly upregulated in fibroblasts when they were stimulated with both TGF beta and ADSC-Exosomes in the culture media. With the ability of ADSC-Exos to inhibit the P38 MAPK signaling pathway, this biomarker can serve as remarkable treatment option for Oral submucous fibrosis. ^26^

In another study, Zhou *et al.* isolated exosomal long non-coding RNA ADAMTS9-AS2 from tissue samples of oral submucous fibrosis and oral squamous cell carcinomas. miRNAs regulated by lncRNA ADAMTS9-AS2 enriched the metabolic pathway, epithelial mesenchymal transition, p13K-Akt pathway and pathways of cancer and enhanced the malignant potential of OSF. ADAMTS9-AS2 plays a crucial role in altering the cell microenvironment during the carcinogenesis process of oral submucous fibrosis and, thus is an ideal marker for early diagnosis of OSCC in oral submucous fibrosis. ^27^

## Exosomes in oral lichen planus

Oral lichen planus is a chronic inflammatory disease of the oral mucosa with an unknown etiology which is characterized by abnormal activity in the T-cell mediated immune response and is also regarded as a “potentially precancerous disorder ^”^. ^28^^,^^29^

Byun *et al.* ^30^ isolated exosomes from the lesions of oral lichen planus and from saliva of 16 patients and eight age-matched normal individuals. The miRNA microarray analysis showed that there were 21 miRNAs that showed a 2-fold increase in the saliva samples of oral lichen planus compared to that in normal individuals. Among all the miRNAs that were identified, hsa-miR4484, hsa-miR1246 and hsa-miR1290 were found to be upregulated in the salivary exosomes of oral lichen planus patients. The miRNA 4484 can target a multitude of genes and initiate their translation into proteins that alter the cellular mechanisms.

## Exosomes in oral cancer

Exosomes derived from oral cancer are saucer-like in shape with a membranous structure. The size of the exosomes in the saliva of oral cancer patients are also larger compared to that of healthy individuals and range from 20-400nm in diameter ^14^ Western blot can detect proteins CD63, Rab 5, CD9 and Alix in the exosomes derived from oral cancer patients. Exosomes derived from oral cancer cell lines contain close to 267 proteins. ^31^

Exosomes are involved in several cellular mechanisms of oral cancers such as tumor growth, invasion, metastasis and chemoresistance. ^9^^,^^32^ Exosomes secreted by neoplastic cells into the tumor microenvironment (TME) play a vital role in tumor growth, invasion and metastasis. ^33^ Exosomes are also found to promote epithelial-mesenchymal transition during the progression of squamous cell carcinomas of the tongue. ^34^^,^^35^ Early-stage tumors release exosomes that contain several tumor markers into saliva and hence they can serve as non-invasive, efficient diagnostic tools. ^5^

Wang *et al.* found that in oral cancer patients with lymphatic metastasis, exosomal laminin 332 was highly expressed. ^36^ Theodoraki *et al.* ^37^ found that exosomal PD-L 1 was associated with the clinical stage of oral cancer. Rabinowitz also found exosomes enriched with miRNA in the tissue samples of tongue oral squamous cell carcinomas. ^38^ The exosomes in saliva from oral cancer patients are larger compared to that of normal exosomes. The density of CD63 in the exosomes of oral cancer patients is markedly increased when compared with normal exosomes. ^39^ Yet the density of the other surface markers of exosomes CD9 and CD81 is significantly reduced in saliva of oral cancer patients when compared with that of normal individuals. ^14^

Exosomes from hypoxic oral squamous cell carcinoma cells are found to deliver the miR-21 to normal OSCC cells. This promotes prometastatic behaviour among tumor cells. ^40^ Exosomes can also spread the invasive potential to non-invasive cells by transferring oncogenic miRNAs. Highly invasive tongue cancer cells can release exosomes containing miR-200-3p that will prevent the expression of CHD 9 and WRN in non-invasive cells and confer a invasive potential to these cells as well. ^41^ Qadir *et al.* found that CEP55 (a centrosomal protein) was present in all the exosomes released from cell lines of Head and Neck Carcinoma cells and absent in the exosomes released from normal oral keratinocytes. ^42^

Liu *et al.* found that oral squamous cell carcinoma cells released chemo resistant exosomes which induce cisplatin resistance in OSCC. These exosomes can upregulate miR-21 and downregulate the expression of phosphate and tensin homolog and programmed cell death. ^43^ Langevi *et al.* found that salivary exosomes expressed elevated levels of miRNA 486-5p in oropharyngeal squamous cell carcinomas compared to controls. ^44^ He *et al.* isolated salivary exosomes from oral squamous cell carcinoma patients and quantified them by NTA and characterized them by TEM and found that salivary exosomes had higher level of miR24-3p in oral squamous cell carcinoma patients. ^45^

Faur *et al.* found that miR-10b-5p, miR-486-5p, miR-24-3p and miR-200a in the exosomes of saliva are the most apparently useful salivary biomarkers of head and neck cancer. ^46^

Exosomal miR-29a-3p derived from OSCC cells can promote proliferation and invasion of OSCC cells by enhancing the polarization of M2-subtype of macrophages, the tumor associated macrophages (TAMs). ^47^ When OSCC cells release exosomes that contain THBS1, they communicate with M1 subtype of macrophages and transform them into TAMs. These TAMs can now promote migration of OSCC cells. ^48^

Zhu *et al.* found that oral cancer cells released exosomes that contained TGF-beta. these exosomes are internalized by natural killer cells in the microenvironment. During the early stages, the proteins in the exosomes enhanced the function of the NK cells. Yet, with a longer incubation time, the TGF-beta gradually inhibited the cellular cytotoxicity of NK cells. The killer function of natural killer cells also decreased. A PANTHER protein class analysis showed that there is salient enrichment of proteins related to localization and adhesion of exosomes to their recipient cells. Then the exosomes fuse with the cell membrane of the recipient cell and transfer their contents in the recipient cell. ^49^

Exosomes derived from oral cancer cell contain NAP1 that enhanced the cytotoxicity of NK cells. ^50^ Exosomes derived from oral squamous cell carcinomas also contain EGFR. ^51^ The EGFR and CD 9 are usually in the same exosome. ^52^ The EGFR secreted by OSCC cells play a vital role in the EMT of epithelial cells. Exosomal EGFR secreted from transformed OSCC play an important role in EMT of normal epithelial cells. Cetuximab is not able to inhibit the EGFR mediated EMT transition of the transformed OSCC cells. ^53^ Metastatic OSCC cells actively secrete chaperone-rich exosomes that are rich in stress resistant protein HSP. ^54^

## Cancer associated fibroblasts

Cancer associated fibroblasts promote tumor progression mainly through actively communicating with cancer cells. CAFs-associated exosomes mediate migration and invasion of OSCC cells. CAF’s derived exosomes exert a stronger effect on upregulation of MMP-3, MMP-9, N-Cadherin and Beta catenin. miR-382-5p are transferred from CAFs to OSCC cells through exosomes. The expression of miR-382-5p in CAFs is elevated by ~3.83 fold compared to that of normal fibroblasts. ^55^ Languino *et al.* showed that exosomes released from cancer-associated fibroblasts transferred TBRII to malignant keratinocyte and activates them to be responsive to the TGF B ligand. ^56^

## Exosomes in salivary gland malignancies

Yang *et al.* in their study found that exosomes loaded with epiregulin from salivary adenoid cystic carcinoma induced epithelial-mesenchymal transition by down regulating the expression of E cadherin. ^57^ Epilegrin-enriched exosomes derived from salivary adenoid cystic carcinoma can also enhance invasion and metastasis of this tumor.

Hou *et al.* found that exosomes-derived salivary adenoid cystic carcinoma 833 cells target the tight junction proteins claudin -1, Zo-1 and beta catenin and enhance migration and invasion of the tumor cells. ^58^

Exosomes are a unique biological entity that play an important role in pathogenesis of potentially malignant oral disorders and head and neck cancers ( Tables 1 and 2).

**Table 1.** Exosomal components in the biology of potentially malignant oral disorders.

Oral leukoplakia		
Exosomal component	Target/tissue	Biological function
Exosomal miRNA 8485 from mesenchymal stem cells of dysplastic oral leukoplakia. ^24^	Dysplastic oral mucosal cell line and human tongue squamous cell carcinoma cell line.	Promotes proliferation, migration, and invasion of dysplastic oral keratinocytes.
Exosomal -miR-185 from Mesenchymal stem cells. ^25^	Buccal lesions in DMBA induces OPMD.	Increased the severity of inflammation. Decreased the number of dysplastic features.

Oral submucous fibrosis		
Exosomal component	Target/tissue	Biological function
Human Adipose-derived mesenchymal stem cell exosome. ^26^	Acts on TGF -β1.	• Reversed the upregulation of Collagen I and Collagen III that is induced by TGF- β1. • Downregulation of COLIA 1 and COL3A I mRNA. • Upregulation of MMP1 and MMP3. • Reversed the upregulation of phosphorylation of p38 that is induced by TGF-.
Exosomal long non-coding RNA ADAMT S 9 -AS2. ^27^	Acts on the p13K-AKT-Signaling pathway. Acts on the Epithelial – Mesenchymal transition pathway. Act on the metabolic pathways.	• Suppresses the malignant transformation of oral submucous fibrosis • Can also inhibit the cell growth, migration and invasion of oral squamous cell carcinoma cells.

Oral Lichen Planus		
Exosomal component	Target/tissue	Biological function
miR-4484. (upregulated). ^30^	Translation of several genes.	Immune reaction or represents a protective mechanism against a pathological stimulus.

**Table 2.** Exosomal components in the biology of head and neck Cancers.

Squamous cell carcinoma		
Exosomal component	Target/Tissue	Biological function
Laminin 332 released from exosomes. ^36^	Over-expressed.	Associated with lymphatic metastasis.
Programmed death ligand 1 and cytotoxic T lymphocyte associated protein 4 present in the exosomes from head and neck cancer cells. ^37^	Induce apoptosis of T cells. Inhibit T cell proliferation.	Promotes tumor cell evasion from the immune system.
Exosomal miR 21. ^40^^,^^43^	Hypoxic oral squamous cell carcinoma cells. Down regulates Phosphate and tensin homolog Programmed cell death.	Prometastatic behaviour of tumor cells. Transfers cisplatin-resistance to non-resistant OSCC cell lines.
Exosomal miR 200-3p. ^41^	Inhibits the translation of CDH, ETNK1, and WRN.	Enhances the invasive potential of non-invasive tumor cells.
Exosomal CEP55. ^42^	ESCRT and ALIX binding region.	Cell division.
Salivary exosomal miRNA-486-5p ^44^	Elevated.	• Screening and diagnosis of oral cancers.
Salivary exosomal miR-24-3p. ^45^	Cell cycle regulatory gene PER 1	• Proliferation of Oral Squamous cell carcinoma cells
OSCC Exosomal derived miR 29a-3p. ^47^	Promotes M2 subtype macrophage polarization. These macrophages produce VEGF, PDGF, cytokines and MMPs.	• Promotes proliferation and invasion of Oral Squamous cell carcinoma • Promotes angiogenesis, cancer growth and metastasis.
OSCC Exosomal THBS1 ^48^	Acts on M1 subtype of Macrophages.	• Transform macrophages into tumor associated macrophages • Regulates the migration of tumor cells.
Exosomal TGF B. ^49^	Reduces the expression of surface receptors NKp30 and NKG2D on natural killer lymphocytes.	• Inhibits the cellular cytotoxicity of natural killer lymphocytes.
Exosomal nuclear Kappa B – activating kinase associated protein 1 (NAP -1). ^50^	Interferon regulatory factor 3-dependent pathway.	• Enhanced the cytotoxicity of natural killer cells.
Exosomal EGFR. ^52^^,^^53^	Epithelial mesenchymal transition of epithelial cells.	• Inhibited by anti-EGFR cetuximab
Exosomal stress resistant protein – heat shock protein. ^54^		• Lymph node metastatic oral squamous cell carcinomas.
Exosomes derived from cancer associated fibroblasts. ^55^	Upregulation of MMP-3, MMP-9, N-Cadherin and Beta Catenin.	• Migration and invasion of CAL 27 lines.
Exosomal miR-382-5p derived from cancer-associated fibroblasts. ^56^	RERG/Ras/ERK pathway?	• Migration and invasion of tumor cells.

Salivary gland neoplasms		
Exosomal component	Target/Tissue	Biological function
Epiregulin-enriched exosomes Derived from adenoid cystic carcinomas. ^57^	Down regulates the expression of E-Cadherin. Enhance the expression of vascular endothelial growth factor receptor 1 in lung endothelial cells.	• Induces epithelial-mesenchymal transition • Enhances invasion and metastasis • Generates a premetastatic niche at the site of future metastasis.
Exosomes derived from Adenoid cystic carcinoma 883 cells. ^58^	Down regulates the proteins of the tight junctions Claudin-1, ZO-1, and catenin.	• Promotes tumor cell migration and invasion.

## Exosomes in oral cancer treatment – drug delivery

Precise targeted delivery of chemotherapeutic drugs is one of the best methods to reduce the toxic side effects of chemotherapy. Exosomes have superior drug delivery properties, such as good stability, allowing them to travel to distant target organs. The hydrophilic core encapsulates water soluble drug molecules. Exosomes are highly biosafe and do not induce an immune response in the body. ^59^^,^^60^ Exosomes can track and monitor tumor progression and drug resistance in real-time giving information on drug heterogeneity. ^61^ A proteome map of human parotid exosomes has also been developed using multi-dimensional protein identification technology, which helps in discovering exosome protein markers related to oral cancer. ^62^

Exosomes can be used as natural drug delivery vehicles for oral cancer. Exosomes can deliver their contents to the target cell by binding to the cell membranes and the receptors present on the surface membrane and then by cytocytosis. ^63^ Exosomal drug loading can either be through three types of a passive methods and four types of active methods. ^64^^–^^70^

One of the passive forms of exosomal drug loading is to transfect the drugs to be loaded into the donor cells and then encapsulate the drugs into the exosomes inside the donor cells. Although the drugs might have a toxic effect on the donor cell, this method is safe and is used in immunotherapeutic treatment for cancer. ^65^ Another method of passive drug loading is to separate and purify the exosomes from the donor cell and mix them with the drug to be loaded. Although the drug loading is slow, the integrity of the exosomal membrane is maintained. ^64^

One of the active methods of loading exosomes with drugs is to prepare a pore in the exosomal membrane by an electrical field and then to allow the therapeutic drug to penetrate the exosomes. By this method, hydrophilic molecules are transported into the hydrophilic core of the exosomes. ^67^ In another active method the exosomes and the drugs are first frozen and then thawed. ^70^

Exosomes can also serve as carriers of therapeutic small molecules, proteins and nuclei acids for therapeutic application in diseases. Exosomes have an amazing advantage of good biocompatibility, almost non-toxic side effects and can be used as a sound drug delivery system. The lipid bilayer membrane of exosomes protects their contents from degradation and destruction, and they are highly stable in circulation. The small size of exosomes gives them the ability to cross various biological barriers such as the blood – brain barrier and reach the target cell or organ. ^63^

Exosomes carry hydrophilic molecules such as miRNAs. An effective way of cancer treatment would be load the exosomes with miRNAs that can inhibit OSCC progress such as miR-1294, miR- 6887-5p or miR-101-3p. It is revealed that Fe3O4 nanoparticles and a constant magnetic field can induce exosomal miR-21-5p upregulation. This method of a combination of nanomaterials and a magnetic field improves the precise localization of drug, drug retention and the drug half-life and reduces the drug dose that is needed and improves drug efficacy. ^71^^,^^72^

The saliva of patients with oral cancer is loaded with proteins related to cancer development and progression. The exosomes of cancer cells express carcinogenic and tumour suppressor miRNAs that are different from that of normal cells. Identification of a combination of exosomal DNA, RNA and proteins can improve the accuracy of exosomal based diagnosis of oral cancer. ^63^ The presence of CD47 on exosomes produces a signal that protects them from phagocytosis. ^73^ Delivering specific DNA, RNA or proteins via exosomes can be an interesting method of treating oral cancers.

A few challenges of employing exosomes in oral cancer diagnosis are that purity and the quality of the exosomes obtained is not high and its time consuming, laborious, costly and at times inefficient. ^72^ Although exosomes are heterogenous in origin and exhibit differential effect on oral cancer progression, it is challenging for vaccine development, and exosomes-based vaccines for oral cancer are in the making. ^74^

## Conclusion

Exosomes are extracellular messengers that transport and exchange valuable molecules across cells and this has a huge influence on the cellular activity of the recipient cell as well as their microenvironment. An astute evaluation of the components of exosomes will provide a greater insight into the underlying molecular mechanism of the disease process. Exosomes are found in abundance in biofluids such as saliva, which can be readily and easily obtained through non-invasive methods. Salivary exosomes are more stable and can be widely used for the diagnosis and early detection of oral cancers. miRNAs in the exosomes provide molecular and genetic information that can assist in the prognosis and for disease monitoring of oral cancer patients. Further investigation in the area of salivary exosomes, will unravel the biological mechanism of exosomes in the tumorigenesis pathway of oral precancers and cancers. Exosomes are very small and can easily pass through various biological barriers, making them very good delivery vectors for therapeutic drugs as well as to selectively induce DNA’s mRNA and miRNAs into targeted cancer cells.

## Data Availability

No data are associated with this article.
