# Supplementary material for: Exosomes: Mediators of cellular communication in potentially malignant oral lesions and head and neck cancers
Source: F1000Res. 2023 Oct 4;12:58. Originally published 2023 Jan 13. [Version 2] doi: 10.12688/f1000research.127368.2 (PMC10696492; doi:10.12688/f1000research.127368.2)
Supplement: Peer review history

# Reviewer report: Reviewer response for version 2

**DOI:** 10.5256/f1000research.156624.r223740

**Published:** 2023-11-30

**Authors:** Jiji George

**Affiliations:** 1 BBD College of Dental Sciences, Lucknow, Uttar Pradesh, India

Monica *et al* has presented a comprehensive report on Exosomes, their origin, role in different disease conditions ranging from immunologic diseases to cancer. The authors have described the molecular characteristics of exosomes and the different methods of identification. The role of various components of the exosomes in the carcinogenic pathway is expounded in the manuscript. The implication of salivary exosomes in oral cancer diagnostics is highlighted. The benefits of drug delivery utilizing exosomes is explained explicitly.

 Strength: The authors have analyzed data from relevant scientific literature and have systematically presented the way in which exosomes are mediators of intercellular communications in premalignant and malignant oral lesions. In addition, the limitations and challenges encountered in the clinical application of exosomes is also described.

 I feel this manuscript can be approved in the present format.

Is the review written in accessible language?

Yes

Are all factual statements correct and adequately supported by citations?

Yes

Are the conclusions drawn appropriate in the context of the current research literature?

Yes

Is the topic of the review discussed comprehensively in the context of the current literature?

Yes

Reviewer Expertise:

Oral cancer

I confirm that I have read this submission and believe that I have an appropriate level of expertise to confirm that it is of an acceptable scientific standard.

# Reviewer report: Reviewer response for version 2

**DOI:** 10.5256/f1000research.156624.r212100

**Published:** 2023-10-25

**Authors:** Ajaz A Bhat

**Affiliations:** 1 Department of Human Genetics-Precision Medicine in Diabetes, obesity and Cancer Research Program, Obesity and Cancer Research Program, Sidra Medicine, Doha, Qatar

I have no further comments to make.

Is the review written in accessible language?

Yes

Are all factual statements correct and adequately supported by citations?

Yes

Are the conclusions drawn appropriate in the context of the current research literature?

Partly

Is the topic of the review discussed comprehensively in the context of the current literature?

Partly

Reviewer Expertise:

Cancer Biology, Immunology, Obesity

I confirm that I have read this submission and believe that I have an appropriate level of expertise to confirm that it is of an acceptable scientific standard.

# Reviewer report: Reviewer response for version 1

**DOI:** 10.5256/f1000research.139868.r177886

**Published:** 2023-06-29

**Authors:** Ajaz A Bhat

**Affiliations:** 1 Department of Human Genetics-Precision Medicine in Diabetes, obesity and Cancer Research Program, Obesity and Cancer Research Program, Sidra Medicine, Doha, Qatar

**Main Findings:**

 The review by Solomon *et al.,* focuses on the role of exosomes, extracellular vesicles that mediate intercellular communication, in the pathogenesis of potentially malignant oral lesions and head and neck cancers. The authors discuss the molecular characteristics of exosomes, highlighting their resilience due to their lipid bilayer membrane and their capacity to transfer biological material, such as miRNA, mRNA, long non-coding RNA, DNA, proteins, and lipids, between cells. They emphasize the importance of exosomes in tumor growth, invasion, and metastasis, and their potential use in diagnosing disease activity. The review also explores the potential of using exosomes as therapeutic vectors due to their size and ability to traverse biological barriers.

**Strengths:**

1. The review provides a comprehensive discussion on the emerging role of exosomes in the context of potentially malignant oral lesions and head and neck cancers, a relatively novel area of research.
2. It delves into the molecular and genetic information carried by exosomes, thereby providing deep insights into their functioning and influence on cancer cells.
3. The review brings to light the potential of exosomes as biomarkers, which is crucial for early diagnosis and personalized treatment strategies.
4. It also explores the potential use of exosomes as therapeutic delivery vectors, which opens a new avenue in the treatment of these types of cancers.

**Limitations:**

1. The title might benefit from minor rephrasing to improve readability. For example: "Exosomes: Mediators of Cellular Communication in Potentially Malignant Oral Lesions and Head and Neck Cancers" or "The Role of Exosomes in Cellular Communication: Implications for Potentially Malignant Oral Lesions and Head and Neck Cancers."
2. Important literature is missing ^1^^,^^2^. Authors should cite these and many other important recent references. Readers might benefit from references to key studies that have shown the importance of exosomes in potentially malignant oral lesions and head and neck cancers.
3. It does not address potential challenges or limitations in utilizing exosomes for diagnosis or therapy, which would provide a more balanced perspective.
4. Authors should have at least one or two figures in the paper.
5. Although the conclusion acknowledges the need for further investigation in the area of salivary exosomes, it could also discuss potential challenges in this line of research or any practical barriers to using exosomes as diagnostic markers or therapeutic vectors.
6. Rather than simply stating the need for more research, the authors could suggest specific areas for future study or the kinds of experiments that could be valuable in this field.

**English Language:**

1. The language is generally clear and well-written. However, there are some phrases that could be reworded for clarity and flow. For instance, "Exosomes provide a multitude of molecular and genetic information and have become valuable indicators of disease activity at the cellular level" could be reworded to "Exosomes, rich in molecular and genetic information, have emerged as valuable indicators of cellular disease activity." There are many such instances.

Is the review written in accessible language?

Yes

Are all factual statements correct and adequately supported by citations?

Yes

Are the conclusions drawn appropriate in the context of the current research literature?

Partly

Is the topic of the review discussed comprehensively in the context of the current literature?

Partly

Reviewer Expertise:

Cancer Biology, Immunology

I confirm that I have read this submission and believe that I have an appropriate level of expertise to confirm that it is of an acceptable scientific standard, however I have significant reservations, as outlined above.

## References

[1] : The role of extracellular vesicles in cancer. *Cell* .2023;186(8) : 10.1016/j.cell.2023.03.010 1610-1626 10.1016/j.cell.2023.03.010 37059067 PMC10484374

[2] : Extracellular vesicles-mediated intercellular communication: roles in the tumor microenvironment and anti-cancer drug resistance. *Mol Cancer* .2019;18(1) : 10.1186/s12943-019-0965-7 55 10.1186/s12943-019-0965-7 30925923 PMC6441157

# Response

**Published:** 2023-09-21

**Authors:** Monica Charlotte Solomon

**Affiliations:** Manipal Academy of Higher education, India

The authors thank the reviewer's for their suggestions and recommendation. Thank you for your time and patience.

1. The title might benefit from minor rephrasing to improve readability. For example: "Exosomes: Mediators of Cellular Communication in Potentially Malignant Oral Lesions and Head and Neck Cancers" or "The Role of Exosomes in Cellular Communication: Implications for Potentially Malignant Oral Lesions and Head and Neck Cancers."  
**The title of the manuscript has been corrected as per the suggestion. **
2. Important literature is missing ^1^ ^,^ ^2^ . Authors should cite these and many other important recent references. Readers might benefit from references to key studies that have shown the importance of exosomes in potentially malignant oral lesions and head and neck cancers.
**The recommended references have been included in the manuscript.**
3. It does not address potential challenges or limitations in utilizing exosomes for diagnosis or therapy, which would provide a more balanced perspective.
**The potential limitation of utilizing exosomes for diagnosis and therapy has been addressed in the manuscript.**
4. Authors should have at least one or two figures in the paper.
**Figures have been included in the manuscript.**
5. Although the conclusion acknowledges the need for further investigation in the area of salivary exosomes, it could also discuss potential challenges in this line of research or any practical barriers to using exosomes as diagnostic markers or therapeutic vectors.
**The practical barriers has been included in the manuscript.**
6. Rather than simply stating the need for more research, the authors could suggest specific areas for future study or the kinds of experiments that could be valuable in this field.
**This has been included in the manuscript.**

# Reviewer report: Reviewer response for version 1

**DOI:** 10.5256/f1000research.139868.r177869

**Published:** 2023-06-26

**Authors:** John W Barrett

**Affiliations:** 1 University of Western Ontario, London, Canada

The authors review the literature surrounding exosomes generally and more specifically the role of exosomes in oral cancer. Although this manuscript is presented as a Review it reads more like a list of observations or facts. The authors provide two organized tables outlining the exosomal components in the biology of malignant disorders (table 1) or head and neck cancers (table 2) but there is overlap. For example, the first example in Table 1 (Oral Disorders) is dysplastic oral mucosal cell line and human tongue squamous cell carcinoma cell line which should properly be in Table 2 (head and neck cancers).

**Minor issues:**

- The first sentence of the Introduction is clearly untrue. I am not sure what the authors meant to say but to state that exosomes were first described in 2014 is incorrect by about 40 years. Exosomes were first described in the 1970s.
- Page 3 TEM section. This section contains a single sentence, and it requires a reference. And in the same section what are the pellets that the authors are referring to?
- Page 3 ELISA section. The authors report the levels of CD63 observed in exosomes from the oral fluids from oral cancer patients compared to normal individuals; however, there is no mention as to whether the differences are statistically significant or how many patients were sampled. Looking at the numbers I would predict that the observed levels overlap and are unlikely to be significantly different. But this may just reflect a small sampling number. Neither is reported but the authors should address this deficiency.
- Page 5 paragraph 2. The authors state that “The size of the exosomes in the saliva of oral cancer patients are also larger compared to that of healthy individuals and range from 20-400nm in diameter”. So what are the sizes of healthy individuals for comparison? They must be extremely small if they are less than 20nm.
- Page 5, last sentence of paragraph 3. “Early-stage tumors release exosomes that contain several tumor markers into saliva and hence they can serve as non-invasive, efficient diagnostic tools." Ok, that is fine but what are these “several tumor markers”? and how can they serve as diagnostic tools?
- On page 5, 5 ^th^ paragraph the authors state that “hypoxic oral squamous cell carcinoma cells are found to deliver the miR-21 to normal OSCC cells." How is this possible? By definition “normal cells” can’t be OSCC (oral squamous cell carcinoma). Please correct and clarify what you mean.
- The authors list several studies that identified numerous miRNAs (9 different miRNAs) in the exosomes of oral cancer patients and only one is shared amongst the reports. A review article should be synthesizing these reports and sifting through the observations to present the reader with the best evidence. That is not clear here. 

Is the review written in accessible language?

Yes

Are all factual statements correct and adequately supported by citations?

Yes

Are the conclusions drawn appropriate in the context of the current research literature?

Yes

Is the topic of the review discussed comprehensively in the context of the current literature?

Partly

Reviewer Expertise:

Translational head and neck cancer biology

I confirm that I have read this submission and believe that I have an appropriate level of expertise to confirm that it is of an acceptable scientific standard, however I have significant reservations, as outlined above.

# Response

**Published:** 2023-09-21

**Authors:** Monica Charlotte Solomon

**Affiliations:** Manipal Academy of Higher education, India

The authors thank the reviewer for their suggestion and recommendations

- The first sentence of the Introduction is clearly untrue. I am not sure what the authors meant to say but to state that exosomes were first described in 2014 is incorrect by about 40 years. Exosomes were first described in the 1970s.
**Thank you, this has been corrected in the manuscript.**
- Page 3 TEM section. This section contains a single sentence, and it requires a reference. And in the same section what are the pellets that the authors are referring to?
**The details of the same has been included in the manuscript.**
- Page 3 ELISA section. The authors report the levels of CD63 observed in exosomes from the oral fluids from oral cancer patients compared to normal individuals; however, there is no mention as to whether the differences are statistically significant or how many patients were sampled. Looking at the numbers I would predict that the observed levels overlap and are unlikely to be significantly different. But this may just reflect a small sampling number. Neither is reported but the authors should address this deficiency.
**The details have been included in the manuscript.**
- Page 5 paragraph 2. The authors state that “The size of the exosomes in the saliva of oral cancer patients are also larger compared to that of healthy individuals and range from 20-400nm in diameter”. So what are the sizes of healthy individuals for comparison? They must be extremely small if they are less than 20nm.
**This has been corrected in the manuscript.**
- Page 5, last sentence of paragraph 3. “Early-stage tumors release exosomes that contain several tumor markers into saliva and hence they can serve as non-invasive, efficient diagnostic tools." Ok, that is fine but what are these “several tumor markers”? and how can they serve as diagnostic tools?
**The details of the same has been included.**
- On page 5, 5 ^th^ paragraph the authors state that “hypoxic oral squamous cell carcinoma cells are found to deliver the miR-21 to normal OSCC cells." How is this possible? By definition “normal cells” can’t be OSCC (oral squamous cell carcinoma). Please correct and clarify what you mean.
**This has been corrected.**
- The authors list several studies that identified numerous miRNAs (9 different miRNAs) in the exosomes of oral cancer patients and only one is shared amongst the reports. A review article should be synthesizing these reports and sifting through the observations to present the reader with the best evidence. That is not clear here. 
**The details are provided.**
